# Transient Postoperative Hemorrhage from Elbow Arthroscopy Portals following Intra-Articular Pentosan Polysulfate Sodium Injection

**DOI:** 10.1155/2022/9428539

**Published:** 2022-09-29

**Authors:** Lindsay A. Parker, Brandan Wustefeld-Janssens, James M. Dundas

**Affiliations:** ^1^University of Missouri Veterinary Health Center, 900 East Campus Drive, Columbia, Missouri 65211, USA; ^2^Atlantic Veterinary College, University of Prince Edward Island, 550 University Avenue, Charlottetown, Prince Edward Island, Canada C1A 4P3; ^3^Colorado State University, Flint Animal Cancer Center, 300 West Drake Road, Fort Collins, Colorado 80523, USA

## Abstract

Four adult, client owned dogs with diagnosed bilateral elbow dysplasia undergoing elbow arthroscopy for removal of fragmented medial coronoid process were identified via a retrospective database search, who also received intra-articular administration of pentosan polysulfate sodium (PPS) (Cartrophen Vet, Biopharm Australia Pty Ltd., Bondi Junction, New South Wales). Dogs had postoperative administration of 5 ml PPS injected into each elbow joint following elbow arthroscopy. Within 1-3 hours of administration, each dog experienced hemorrhage from arthroscopy incisions that was determined to be independent of surgical trauma given lack of hemorrhage intraoperatively. Pressure bandages were placed, and the hemorrhage and elevated coagulation parameters resolved 12-18 hours following intra-articular injection. No further intervention was required, and the dogs were discharged 20-26 hours postoperatively. The purpose of this case series is to describe 4 dogs who experienced transient and focal hemorrhage following off-label intra-articular administration of pentosan polysulfate sodium (PPS). While this case series is limited due to small number of cases, results following bilateral, intra-articular injection of PPS support a transient systemic coagulopathy. Though this report represents administration of PSS via a route and at doses beyond that recommended on the label, results suggest that administration of PSS in the manner described in this report should be avoided.

## 1. Introduction

Pentosan polysulfate sodium (PPS) is of plant polysaccharide origin and is classified as a disease-modifying osteoarthritis drug which aims to alleviate clinical signs associated with osteoarthritis by stimulating cartilage and synovial fluid production and stem cell activity, increasing subchondral bone perfusion, and acting as an anti-inflammatory [1, 2]. Known commonly by its brand name, Cartrophen, this injectable medication is labeled for subcutaneous administration at a dose of 3 mg/kg to manage osteoarthritis signs. PPS has a mild anticoagulant effect by stimulating tissue plasminogen release, which activates the fibrinolytic system [1, 3]. Despite this side effect, PPS is not known to result in systemic alterations in the clotting cascade. The objective of this case series was to document the reported adverse effects of off-label intra-articular administration of PPS that resulted in focal hemorrhage due to transient coagulopathy in a clinical setting.

## 2. Case Presentation

A retrospective search was performed between April 2018 and June 2019 through the university record database to identify dogs who had previously undergone elbow arthroscopy. Four dogs who underwent bilateral elbow arthroscopy and each had 5 ml of pentosan polysulfate sodium administered intra-articularly were identified and are discussed in this case series. Cases that did not receive intra-articular PPS were excluded.

### 2.1. Case 1

A one-year-old male neutered, 83 kg Irish Wolfhound presented in April 2018 for chronic bilateral thoracic limb lameness.

The dog previously had been treated for left thoracic limb lameness caused by un-united anconeal process (UAP) by excision of the process at 8 months of age. Arthroscopic evaluation of the joints had revealed no other abnormalities. The left thoracic limb lameness recurred in December 2017 (9 months post-op). The lameness did not improve with acupuncture, gabapentin 2400 mg (28.9 mg/kg) (Teva Canada Ltd., Toronto, Ontario), tramadol 700 mg (8.4 mg/kg) (Tramadol hydrochloride tablets USP Teva Canada Ltd., Toronto, Ontario), or cannabidiol. The manufacturer and concentration of cannabidiol were not recorded in the medical record. Radiographs revealed progression of degenerative joint disease of the elbows, and specialty surgery referral was elected.

On presentation, a bilateral thoracic limb lameness and decreased elbow range of motion were noted, both worse on the left. Computed tomography (CT) of the elbows was performed, which indicated degenerative joint disease, incongruity, and fragmentation of the medial coronoid process, all worse in the left elbow. Arthrocentesis of both elbows and the right carpus revealed only mild inflammatory changes in the left elbow. Aerobic culture of joint fluid was negative for bacterial growth. Bilateral elbow arthroscopy was recommended to remove the diseased medial coronoid processes. Preoperative complete blood count (CBC) and serum biochemistry profile revealed a normal CBC and mildly elevated alkaline phosphatase (282 U/L; 18-113 U/L). Preoperative packed cell volume (PCV) was 39% (reference range: 37-55%), and total solids (TS) were 5.8 g/100 ml (reference range: 5.4-7.6 g/100 ml).

**Figure 1:**
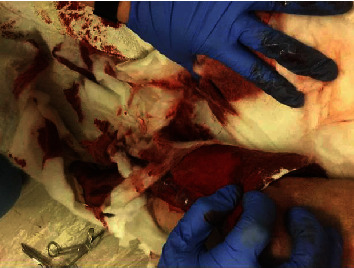
Case 1: dog hemorrhage post-intra-articular PPS administration.

The dog was anesthetized for bilateral elbow arthroscopy performed through two standard arthroscopy portals [4]. Fragmented coronoid process (FMCP) was identified and debrided bilaterally, and intra-operative hemorrhage was minimal, as anticipated. Following closure of the arthroscopy portals, 5 ml of PPS was injected intra-articularly into each elbow, equivalent to a total dose of 12.0 mg/kg for this dog. Approximately 1 hr following administration of PPS, the dog experienced persistent hemorrhage from arthroscopy incisions (Figure 1). Buccal mucosal bleeding time (BMBT) was performed 5.5 hours post-op and was normal at 3 minutes, 8 seconds (reference range: 2-4 minutes [5]). Modified Robert Jones compression bandages were applied and were changed 6 times over the following 9.5 hours due to recurrent strikethrough. PCV and TS were assessed 18 hours postoperatively and were normal at 42% and 6.8 g/100 ml, respectively. Prothrombin time (PT) was normal at 6.5 seconds (reference range: 6.1-8.0 seconds), while activated partial thromboplastin time (aPTT) was found to be mildly prolonged at 16 seconds (reference range: 8.8-14.8 seconds). Hemorrhage was clinically resolved by the following morning, approximately 18 hours following PPS administration. The dog was discharged to the owner for continued postoperative care, and no further hemorrhage was reported. It was estimated that the dog lost approximately 200 ml of blood, equivalent to approximately 3% of the dogs estimated blood volume, through the arthroscopy portal incisions.

### 2.2. Case 2

An 8 month, male intact 28 kg Labrador Retriever presented in May 2018 for a left thoracic limb lameness which had not resolved with medical management and meloxicam (0.1 mg/kg q24 h) (Metacam; Boehringer Ingelheim (Canada), Burlington, Ontario). On presentation, the dog was noted to be subjectively mild to moderately lame with the left thoracic limb. Pain was elicited on compression of the medial compartment of the elbow by supination of the antebrachium (left more severe than right). CT imaging of the elbows revealed mild degenerative joint disease on the left side, with blunting and thickening of the medial coronoid regions bilaterally, without fragmentation. Bilateral elbow arthroscopy was recommended.

Preoperative CBC and serum biochemistry profile were normal, including PCV of 40% and TS of 5.7 g/100 ml. The dog was anesthetized and underwent bilateral elbow arthroscopy using standard medial arthroscopy ports. During arthroscopy, the region of the medial coronoid processes was debrided to bleeding and subchondral bone bilaterally. Following completion of bilateral elbow arthroscopy, 5 ml of PPS was injected intra-articularly into each elbow (total dose 35.7 mg/kg). During the initial recovery period postoperatively (approximately 3 hours following PPS), the dog developed persistent hemorrhage from the elbow arthroscopy incisions. The hemorrhage was managed with modified Robert-Jones compression bandages that were changed as needed overnight due to recurrent strikethrough. By 14 hours following initiation of hemorrhage, it had resolved from the left arthroscopy site, and there was mild, persistent hemorrhage from the right arthroscopy site. Right arthroscopy hemorrhage was resolved by 17 hours. The amount of total estimated blood loss was not documented, but the hemorrhage resulted in bandage strikethrough twice overnight. The dog was discharged to the owner one day post-op. No further hemorrhage occurred.

### 2.3. Case 3

A one-year-old, female spayed 37 kg Great Pyrenees crossbreed presented April 2019 for a one-month history of left thoracic limb lameness. The lameness had failed to improve with medical management and meloxicam (0.1 mg/kg q24h). Radiographs taken by the family veterinarian showed changes consistent with FMCP of both elbows. On presentation, the dog was found to be subjectively mildly lame in the left thoracic limb at a walking/trotting gait. Discomfort on palpation subjectively reduced range of motion, and crepitus was noted in both elbows.

Sedated CT of the elbows revealed bilateral elbow dysplasia. The left medial coronoid process was confirmed to be fragmented, and the right had an associated fissure. Additionally, there was mild bilateral degenerative joint disease changes and subtle to mild bilateral humeroulnar incongruity. Bilateral elbow arthroscopy was recommended to debride the diseased processes.

**Figure 2:**
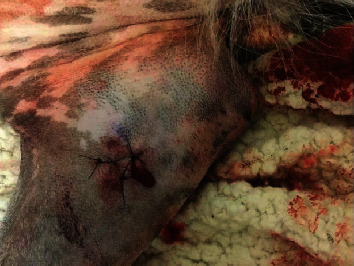
Case 3: dog hemorrhage post-intra-articular PPS administration.

Preoperative CBC and serum biochemistry profile were normal, including PCV of 41% and TS of 5.6 g/100 ml. The dog was anesthetized for bilateral elbow arthroscopy. Medial coronoid disease was identified in both elbows, and the coronoid processes and subchondral bone were debrided bilaterally. Upon completion of the arthroscopy procedures, 5 ml of PPS was administered intra-articularly, equivalent to a total dose of 27 mg/kg. Approximately 1 hour following PPS administration, the dog was found to have active hemorrhage occurring from each arthroscopy portal site (Figure 2). Bilateral modified Robert-Jones compression bandages were placed, which were changed once overnight due to strikethrough. Approximately 16 hrs following PPS administration, the hemorrhage had resolved. PCV and TS were assessed 18 hours postoperatively and were normal at 38% and 5.2 g/100 ml, respectively. The dog was discharged one day post-op. The amount of total estimated blood loss was not documented.

### 2.4. Case 4

An 8-month-old, male intact, 39.6 kg Labrador Retriever presented June 2019 for a four-month duration of right thoracic limb lameness. Radiographs of the elbows taken by the family veterinarian revealed irregularity of the medial coronoid process bilaterally.

On examination, the dog was found to be subjectively moderately lame on the right thoracic limb. The right elbow was held in abduction at rest, and pain was elicited through range of motion. Subjectively, moderate to severe elbow effusion was present bilaterally, and the remainder of the dog's examination was within normal limits.

Preoperative CBC and serum biochemistry profile were within normal limits, including PCV of 42% and TS of 6.4 g/100 ml. Preoperative PT and aPTT were normal with values of 9.4 and 10.9, respectively. CT of the thoracic limbs showed moderate bilateral humeroulnar incongruity and bilateral FMCP with secondary degenerative joint changes (right more severe than the left). BMBT performed under sedation was normal at 1 minute and 41 seconds. Bilateral elbow arthroscopy was recommended to debride the FMCPs.

**Figure 3:**
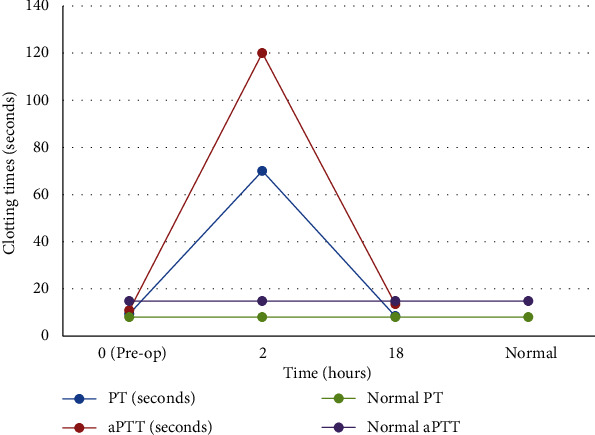
Coagulation parameters (PT and aPTT) following intra-articular PPS administration in case 4.

The dog was anesthetized for bilateral arthroscopy using standardized technique with medially placed ports. FMCPs were identified bilaterally and debrided. Upon completion of the arthroscopy, 5 ml of PPS was administered intra-articularly into each elbow, at a total dose of 25.2 mg/kg PPS. Approximately 2 hours following administration of PPS, the dog was found to have moderate swelling over the medial aspect of the elbows bilaterally. On palpation of these swollen areas, hemorrhagic fluid was expressed through the arthroscopy incisions bilaterally. Repeat coagulation testing was performed, which revealed a marked prolongation of PT/aPTT parameters; PT was greater than 70 seconds, and aPTT was greater than 120 seconds. Modified Robert-Jones compression bandages were placed and did not require replacement overnight. Hemorrhage resolved by 19 hrs post-PPS administration and no additional hemorrhage from the incisions was noted. PT and aPTT were reassessed 18 hours after PPS administration and had significantly improved; PT was mildly prolonged at 8.4 seconds, and aPTT was normal at 13.5 seconds. The changes in PT and aPTT case 4 are demonstrated in Figure 3. The estimated total volume of blood loss was not documented. The dog was discharged home to the owner to continue medical management of bilateral elbow dysplasia.

## 3. Discussion

Canine elbow dysplasia is a multifactorial, developmental orthopedic condition most prevalent in large and giant breed, male dogs that results in lameness and degenerative joint disease (DJD). It is composed of several separate but related conditions including fragmented medial coronoid process (FMCP), elbow incongruity, ununited anconeal process, and osteochrondrosis of the distal, medial humeral condyle (OCD) [6]. Arthroscopic removal of the fragment and debridement of the wound bed is generally recommended for OCD and FMCP, particularly early in the disease process before severe DJD sets in. Treatment 50 to 100% of dogs will have functional improvement, even with worsening radiographic osteoarthritis signs [7]. Following surgery, long-term medical treatment is recommended. Current treatment options include weight management, oral analgesics, nutraceuticals, activity restriction, physiotherapy, and intra-articular injections. Intra-articular injections may include stem cells, platelet-rich-plasma, steroids, glucosamine/chondroitin/hyaluronic acid, and or interleukin-1 receptor antagonist protein [6].

Cartrophen, or pentosan polysulfate sodium (PPS), is classified as a disease-modifying osteoarthritis drug and is an injectable polysaccharide of plant origin [1, 2]. Cartrophen aids in treating signs associated with osteoarthritis by focusing on rebalancing intra-articular metabolic processes by stimulating cartilage production, improving quality and quantity of synovial fluid, stimulating stem cell activity and cartilage cell differentiation, increasing blood supply to subchondral bone, providing strong anti-inflammatory response, inhibiting cartilage degradation enzymes, increasing production of free radical scavenging enzymes, and stimulating growth enzymes and modulates several noxious substances [1, 2]. Treatment is given at a 3 mg/kg subcutaneous dose every 5-7 days for 4 treatments total, then on an as needed basis thereafter [1]. It is recommended that a yearly full course is required at a minimum for best efficacy [1]. The administration of intra-articular PPS was off-label use of this drug, and this case series documents the adverse side effects of this route.

PPS was selected to be administered intra-articularly by the primary clinician to promote overall health of the synovial fluid at the primary joint of concern, which was shown to improve biochemical markers of osteoarthritis disease activity in a double-blind study by Rasaratnam et al. in human patients with osteoarthris [8]. Additionally, when injected intra-articularly 2 to 6 times into rheumatoid arthritic joints in human patients, the molecular weight of hyaluronan increased by a median of 69% [9]. These results were similarly replicated in another study where 4 treatments of weekly PPS intra-articular joint injections increased the concentration of hyaluronan by an average of 58.4% in patients with osteoarthritis [10]. There is little evidence regarding the risks associated with intra-articular administration of PPS in human or veterinary literature. One veterinary source reported that intra-articular injection of PPS may predispose patients to septic arthritis [11]. However, this statement was based on an equine research paper of a combined pentosan polysulfate-glucosamine intra-articular injection, which identified a transient increase in synovial neutrophilic inflammation that resolved without intervention and did not result in septic arthritis as the previous source implied [12]. Furthermore, the study deemed intra-articular administration of the pentosan polysulfate-glucosamine combination resulted in a mild inflammatory synovitis that was not substantially different to saline injection [12]. Moreover, none of these studies described hemorrhage as a complication from the injected joints. Intra-articular administration of PPS is considered extra-label-drug usage (ELDU). Off-label drug usage is not uncommon in several countries, including Canada, and is considered acceptable by the Canadian Veterinary Medical Association if there is a valid veterinarian-client relationship explaining the off-label usage of this drug and potential for adverse side effects [13]. All clients consented to intra-articular administration of PPS.

In this case series, intra-articular PPS was associated with class 1 postoperative hemorrhage, defined as up to 15% of blood volume [14]. None of the cases was significant hemorrhage observed at the time of surgery. Both primary and secondary clotting abnormalities were investigated in these dogs. Buccal mucosal bleeding time (BMBT) was normal in cases 1 and 4, which suggests that platelet function was appropriate in these two dogs. Though BMBT was not performed on each dog, it was done on case 1 at 6 hours postoperatively, while clinical hemorrhage was active. This is further confirmation that PSS^a^ does not affect platelet function. Substantial prolongation of aPTT and PT in case 4 suggests that PPS is capable of significantly affecting secondary hemostasis. Whether this is due to the intra-articular route of administration, the doses administered (12.0–35.7 mg/kg), or both, is unclear. It is worth noting that administration of PPS in this manner is off-label and in excess of the dosing guidelines given on the package. The weight discrepancies between dogs resulted in an administered dose range of 12.0 mg/kg to 35.7 mg/kg which did not subjectively change the severity of the hemorrhage. The administered dose was 4.0-11.9 times the recommended subcutaneous dose. Fortunately, hemorrhage was transient, localized to site of administration, and none of the dog's required intervention past placement of compression bandages.

A few studies have reported on potential side effects of PPS. Cartrophen dose response studies have demonstrated that dosages of 1 mg/kg and 5 mg/kg were not as effective [15]. Higher doses did not reportedly result in a toxic effect [15]. Another study reported suspected adverse reactions (SAR) attributed to Cartrophen Vet administration in the UK^16^. Over 8 years, the number of reported adverse effects was 161; for an adverse effect rate of 0.058% on a per-course basis, only 51% of those were classified as “likely” or “probably” related to Cartrophen Vet administration, leading adverse effects of Cartrophen Vet to be considered “overall mild and transitory” [16]. Of the “likely” or “probably” linked events, only 15 were hematologic abnormalities including anemia, thrombocytopenia, and/or hemorrhage. There is one report in human medicine describing hemorrhage suspected secondary to Elmiron; Alza Pharmaceuticals, Mountain View, California; however, this patient had also sustained a carotid artery puncture during attempted placement of a right internal jugular catheter and significant comorbidities including ulcerative colitis and associated severe malnutrition that may have compounded coagulopathy findings [17, 18].

Other studies have specifically investigated changes in coagulation times related to Cartrophen Vet. Data on file on the Biopharm Australia website shows that when a 3 mg/kg weekly dose was administered subcutaneously for 12 treatments, PT and aPTT were increased at 2 hours postadministration. Values returned to normal by 8 hours postadministration [1]. This transient effect on PT and aPTT is consistent with the normal coagulation values in cases 1 and 4 at 18 hours postoperatively. Data available from Cartrophen Vet also shows that increases in PT and aPTT were noted at 24 hours following administration of Cartrophen Vet at 30 mg/kg for 12 weeks. There were no reported intolerance reactions, including hematoma formation or evidence of systemic bleeding, at any dose. With this drug's pharmacological profile, hemorrhage would not be expected in an animal with normal hemostasis and therefore presents a low risk of hemorrhage despite transient effects on clotting parameters [1]. PT and aPTT were tested preoperatively in case 4 and were normal, confirming that this dog had no hemostatic abnormalities; yet, moderate hemorrhage still occurred—a fact which contradicts the statement on the website. Figure 3 helps to illustrate the transient, but clinically apparent, changes in coagulation profile in case 4. Though whether this discrepancy is due to the route of administration, the dosage, or both, is unclear, but there is evidence to suggest increased doses of PPS are at least partly to blame. In a rabbit ear bleeding model, a 5 mg/kg intravenous dose of PPS resulted in a “minimal, but significant hemorrhage effect” when rabbits were given a triple overdose [19].

The mechanism behind the effect of PPS on hemostasis has been investigated in several studies. PPS stimulates the release of tissue plasminogen activator from endothelium and therefore acts as a fibrinolytic system activaton [1, 19]. This produces a mild anticoagulant effect of PPS, of 1/6 to 1/10^th^ the potency of heparin [1]. Cartrophen states that PPS “does not exhibit a global alteration of the blood clotting system”, unlike heparin [1]. In this case series, we demonstrate that intra-articular administration of PPS does appear to have an effect on the systemic coagulation system when administered intra-articularly. Marsh and Gaffney investigated the effect that pentosan polysulfate (SP54) has on the fibrinolytic enzyme system using a rat experimental model [20]. They concluded that pentosan polysulfate (SP54) resulted in a transient increase in blood fibrinolysis, and that a dose of 10 mg/kg given 90 minutes following thrombus formation was sufficient to achieve thrombolysis [20]. PPS also appears to suppress release of tumor necrosis factor alpha from activated monocytes, which decreases release of plasminogen activator inhibitor [19, 21].

Consultation with the American Society for the Prevention of Cruelty to Animals (ASPCA) Animal Poison Control Center revealed that this was the first intra-articular PPS administration to be reported to them. The ASPCA Animal Poison Control Center did not have access to LD-50 dose for PPS, and we were unable to recover this data through our research. The ASPCA consultant felt that the hemorrhagic fluid and associated prolonged coagulation times were consistent with a high dose of PPS, whether it would be administered intra-articularly, intramuscularly, or subcutaneously. The induced coagulopathy appears to be dose-dependent and therefore, we may not have observed the joint effusion should the dogs in the case series have been given a smaller dose. Per the ASPCA toxicologist, with doses above 18 mg/kg via any administration route, prolonged PT and aPTT would be expected. However, even if associated signs develop, there is a low risk that they would be life threatening or serious.

There are limitations in this case series including its retrospective nature, case number, and inconsistent coagulation parameter testing. However, the faculty surgeon, procedure, and dose administration of 5 ml PPS per elbow was the same for all dogs, and each experienced localized hemorrhage from the arthroscopic sites. Given the similarities between the cases, we feel it is reasonable to extrapolate the coagulation parameters assessed in case 4 to all cases reported here. Alternatively, we could have assessed lactate concentrations as this has been reported to be associated with increased fibrinolysis [22].

Our findings suggest that PPS can result in systemic coagulopathy and localized hemorrhage. This hemorrhage spontaneously resolved 12-18 hours after administration. It may be beneficial to monitor coagulation profiles during PPS therapy, even when administered in the on-label route, to ensure that dogs are not experiencing transient coagulopathies that go unreported. Due to the previously described fibrinolytic nature of PPS, treatment with aminocaproic acid could be beneficial to stabilizing the fibrin clot and resolving hemorrhage if it occurs. Aminocaproic acid is an antifibrinolytic agent that is a synthetic lysine analogue which blocks the lysine binding site of plasminogen, to inhibit its conversion to plasmin and subsequently stabilizes a thrombin clot by allowing fibrinogen to accumulate and prevent onset of fibrinolysis [3].

In this case series, we described an apparently transient coagulopathy following off-label intra-articular PPS administration, resulting in focal hemorrhage from arthroscopy sites. As noted previously, PPS is generally considered a safe medication, and our results were likely a result of the off-label route of administration and/or the high dosage administered. Therefore, off-label administration of PPS should be used with extreme caution, and intra-articular administration should be avoided in dogs.
